# Cloud-Based Breast Cancer Prediction Empowered with Soft Computing Approaches

**DOI:** 10.1155/2020/8017496

**Published:** 2020-05-18

**Authors:** Farrukh Khan, Muhammad Adnan Khan, Sagheer Abbas, Atifa Athar, Shahan Yamin Siddiqui, Abdul Hannan Khan, Muhammad Anwaar Saeed, Muhammad Hussain

**Affiliations:** ^1^Department of Computer Science, National College of Business Administration and Economics, Lahore, Pakistan; ^2^Department of Computer Science, Lahore Institute of Science and Technology, Lahore, Pakistan; ^3^Department of Computer Science, Lahore Garrison University, Lahore, Pakistan; ^4^Department of Computer Science, CUI, Lahore Campus, Pakistan; ^5^Department of Computer Science, Minhaj University, Lahore, Pakistan; ^6^Department of Computer Science, Virtual University, Islamabad, Pakistan

## Abstract

The developing countries are still starving for the betterment of health sector. The disease commonly found among the women is breast cancer, and past researches have proven results that if the cancer is detected at a very early stage, the chances to overcome the disease are higher than the disease treated or detected at a later stage. This article proposed cloud-based intelligent *BCP-T1F-SVM* with 2 variations/models like *BCP-T1F* and *BCP-SVM*. The proposed *BCP-T1F-SVM* system has employed two main soft computing algorithms. The proposed BCP-T1F-SVM expert system specifically defines the stage and the type of cancer a person is suffering from. Expert system will elaborate the grievous stages of the cancer, to which extent a patient has suffered. The proposed BCP-SVM gives the higher precision of the proposed breast cancer detection model. In the limelight of breast cancer, the proposed BCP-T1F-SVM expert system gives out the higher precision rate. The proposed BCP-T1F expert system is being employed in the diagnosis of breast cancer at an initial stage. Taking different stages of cancer into account, breast cancer is being dealt by BCP-T1F expert system. The calculations and the evaluation done in this research have revealed that *BCP-SVM* is better than *BCP-T1F*. The *BCP-T1F* concludes out the 96.56 percentage accuracy, whereas the *BCP-SVM* gives accuracy of 97.06 percentage. The above unleashed research is wrapped up with the conclusion that *BCP-SVM* is better than the *BCP-T1F*. The opinions have been recommended by the medical expertise of Sheikh Zayed Hospital Lahore, Pakistan, and Cavan General Hospital, Lisdaran, Cavan, Ireland.

## 1. Introduction

Apparently, the diagnosis and the scrutiny of the breast cancer disease have always been a decisive and critical one in the regard of medical department. The cancerous lumps form in a particular area of the body when the human cells begin to produce rapidly beyond the expected limit. The cancerous lumps which are also termed as tumors are comprised of two kinds: one is the benign and the other is malignant. Breast cancer is considered as a lump that is formed in the breast cells; when these cells begin to grow irregularly in a human body, it results in flaking and redness of the breast. The cancer is still considered as the undiagnosed and untreated disease in various parts of the world. The questions arise, why the cancer still has the strong roots in patients? Why still breast cancer remains undiagnosed amongst the women? Why the ratio of mortality among women due to breast cancer is still constant? The breast cancer should be diagnosed at an early stage so that the condition does not persist and many lives could be saved. If it gets diagnosed initially, then the chances to overcome the disease are certainly higher. Women are being impinged by this disease commonly. So if cancer remains undiagnosed, then it may lead to death [1, 2]. The risk factors evolve because of which breast cancer is induced may be the genetic reasons, alcohol intake, dense tissues in breast, radiation exposure, age, and so on. Since 1989, the survival rate amongst masses has been immensely improved due to modern technologies introduced in screening and treatment. The recently conducted researches have shown that, in 2017, 252,710 women were diagnosed with this disease; approximately 40,610 according to the statistics were more likely to die from breast cancer. The radical steps in reducing the risk factors of this disease are the awareness of the induction causes of the breast cancer. The early symptoms of cancer and screening can lessen the factors of cancer infringing masses [3].

The physical diagnosis of this mortal disease involves the breast exam, imaging tests, biopsy, blood tests, and so on. The initial blood marker tests which are CA 15.3, TRU-QUANT, CA 125, CEA, and so on are done before the treatment of this cancer. The blood marker tests work as an initial indicator for this disease; in determining the breast cancer, currently there are four main methods which are employed in differentiating between the malignant lump and benign lump: biopsy, fine needle aspiration, mammography, and MRI [4, 5]. The proposed methodology has employed the two algorithms to detect an ailment efficiently. The Mamdani inference system is used among the recent researches to detect a particular kind of disease; this proposed research has compiled the previously done evaluations in comparison to this proposed methodology [6]. Mammography determines the incomplete diagnosis of the infection; if the infection results conclude out to be negative, the infection turns out as benign. Mammography determines whether there is any lump, no lump, or cancerous lump. Mammography concludes the severity level and the type of cancer is measured with the help of biopsy gold test. The biopsy gold test (type) will also determine the benign, ductal carcinoma, invasive lobular carcinoma, inflammatory breast disease, and lobular carcinoma [7, 8].

The recent researches show that, in 2017, 252,710 women were diagnosed with this disease; approximately 40,610 which were more likely to die from breast cancer [1, 9, 10]. The physical diagnosis involves the breast exam, imaging tests, biopsy, blood tests, and so on. In regard of determining the breast cancer, currently there are four main methods which are used in differentiating between the malignant lump and benign lump: biopsy, fine needle aspiration, mammography, and MRI [2, 3, 11].

In this research, fuzzy logic and SVM are used to find out the breast cancer by using different statistical measures such as accuracy, miss rate, specificity, sensitivity, false-positive value, false-negative value, likelihood ratio positive, likelihood ratio negative, positive prediction value, and negative prediction values. With the help of these matrices, breast cancer can be found more accurately as compared with the previous literature.

## 2. Literature Review

To avoid the faulty reasoning processes, errors, continuous failures, lack of knowledge, and failures in rules of logics in detection of tumors, researchers have started to advance new methodologies, models, and tools. Bearing a clear aim, various systems and proposed models have been put forward in successful identification of breast cancer. Among women in America at the rate of one in three cancers approximately, breast cancer is the most frequently diagnosed cancer among the women [6, 12, 13]. Surgical biopsies confirm malignancy with high level of sensitivity but are considered costly and can affect patient's psychology as well. This research demonstrates novel approach by using morphological operators and clustering algorithm fuzzy c-m to identify malignant lump in mammography automatically [6, 14–17]. In the article, initial identification of tumor, fuzzy system's various applications, and algorithms have been proposed [18, 19]. In some previous studies on FDTs, proposed approaches focus on modification of decision tree pruning algorithm and require fuzzy parameters to be set by domain experts. We opted to fuzzify already generated decision tree nodes to relax the sharp decision boundaries. A similar kind of approach is employed in [20, 21].

Fuzzy logic has been rarely used in cancer prognosis. Being noncrisp, it can act as a natural ally of a physician in prognostic decision-making process [22]. In the recent researches, we have surveyed various types of research scenarios [21–23]; in the prognosis of cancer, the applications of various cases of machine learning techniques are contributing towards the advanced researches. Some of the basic trends which are encountered for the motivation of experiments include the following: the fuzzy logic has been used in the diagnosis of cancer rarely. Aiming for clear interpretation of a particular type of disease physicians, using “Black Box” models, approximately 70% of all researches reported making use of neural networks. The majority of the manuscripts used machine learning techniques independently without considering potential in the discussed manuscripts to cope up with each other in a hybrid model. Lack of attention is paid to data size. Victor gives one solution to computerized tool used for diagnosis of breast cancer. The fact is that fuzzy logic can substantially assist in diagnosis of breast cancer is being put forward in this paper [24–26].

Diagnosis of breast cancer is through fuzzy clustering with partial supervision [27]. ARTMAP approach gives 97.2% accuracy, which represents the one-way approach in the neural networks for the diagnosis of breast cancer [28]. Classification exactness of over 95% was professed to be accomplished by utilizing little MIAS database; the proposed framework is being acknowledged for the findings of bosom malignancy dependent on outrageous learning machine [29–32].

Resisting a technique for classification of mammogram that is comprised of 4 phases, preprocessing stage utilized middle filter to upgrade nature of picture and to expel clamor from the picture. To check the variation from the norm of the mammograms, ANN classifier was utilized to group the picture into fitting class. Affectability, specificity, and precision asserted in the work were 72.72%, 93.6%, and 88.66% [18, 33].

A framework for the findings of bosom malignancy dependent through feedforward networks was proposed. Prepreparing was done in two-phase foundation and second was evacuating pectoral muscle. Hough change strategy was utilized for ROI. An aggregate of 32 dark dimensions and surface highlights is separated from mammograms. Precision asserted by utilizing smaller than normal MIAS database was 94.06% [34].

A few systems have been sent to anticipate and perceive significant example for breast malignancy analysis. Data mining further categorizes the different methods of the decision tree, ANN, RIPPERS classifier, and Support Vector Machine (SVM) to make a quick explanation and survey of the dataset regarding the cardiovascular disease. The explanation used the consideration and comparison of the performance of the techniques which encompasses accuracy, sensitivity, specificity, error rate, true positive rate, and false positive rate [35, 36].

Computational intelligence approaches like fuzzy system [37–39], neural network [40], and swarm intelligence [41] and evolutionary computing [42] like genetic algorithm [43, 44], DE, Island GA [45], Island DE [46, 47], classifier [48], and SVM [49] are strong candidate solutions in the field of smart city [50], wireless communication, and so on.

## 3. Proposed System Model Methodology

**Figure 1:**
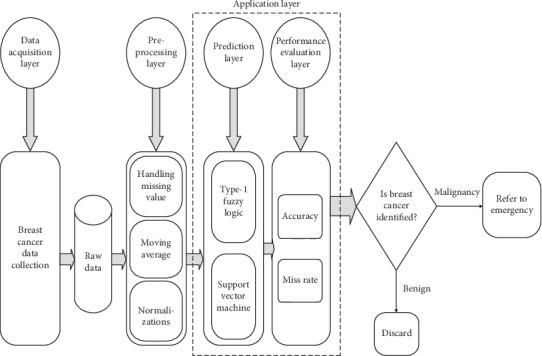
Proposed intelligent breast cancer prediction model for BCP-T1F-SVM expert system.

The following methodology has been elaborated in Figure 1. First layer is the data acquisition layer which follows up with the data collection of breast cancer. The raw data attained through the collection of breast cancer is then fed into the preprocessing layer. The preprocessing layer is a criterion to handle the missing values amongst the raw data; furthermore, moving average and normalizations are being done in the preprocessing layer. The omissions and errors are being lessened through the standard portable. Then after the completion of the previous layers, the preprocessing layer then jumps on to the application layer. The application layer is comprised of the prediction layer and the performance evaluation layer. The prediction layer specifically focuses on the two algorithms which are employed to determine the indispensable types of breast cancer through type-1 fuzzy logic and SVM just points out that something is fishy or not; that is, a person is suffering from the disease or not. The two algorithms which aided the application layer are shown in Figure 1. Type-1 fuzzy logic is an enabled system used to get accurate results from big data. The performance evaluation layer calculates the accuracy and miss rate. Type-1 fuzzy logic constitutes of logical rules and these rules can be defined easily by the help of a medical expert. Type-1 fuzzy logic rules are applied on inputs of fuzzy sets and then converted it into a fuzzy output. In this research, input variables are used to propose a system to diagnose the particular disease which is cardiac by using a fuzzy logic model. For the detection of cardiac disease, Support Vector Machine (SVM) is a model that provides computational results which depend upon the structure and biological functions of neural networks. In the prediction layer, Support Vector Machine is used to find out the breast cancer, and in the performance layer, it is used to evaluate the results produced by the prediction layer performance. The whole system process is shown in Figure 1, in which the data acquisition layer comprises the parameters of input. In this model, they will go for the neural system, where a trained algorithm is used to estimate breast cancer. At the industrial level, SVM is utilized and it gives accurate results. SVM includes several neurons that are specifically organized. Neurons and influences among them are essential parts of an SVM. Neurons have handling features that cooperate to overcome an issue. This layer is used to examine the breast cancer on the basis of thirty input parameters, which is termed as the scientific study of the models that are statistical in nature and constitute algorithms which computer systems employ to perform a certain type of task with greater precision and as certainty. In the performance evaluation layer, precision and miss rate are determined. In the decisive area, the conclusion is made whether the breast cancer is identified or not.

### 3.1. Fuzzy System Methodology

**Figure 2:**
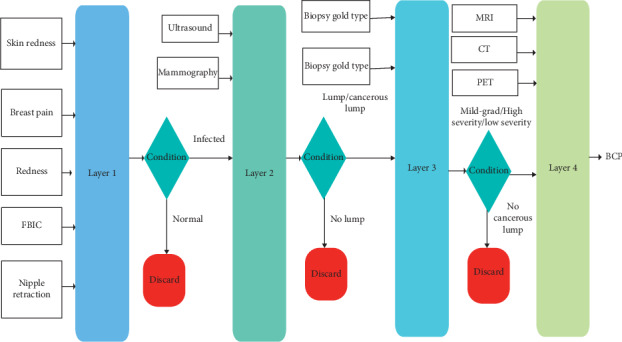
Proposed BCP-T1F expert system methodology.

Our proposed model breast cancer prediction (BCP), multilayered Mamdani fuzzy type-1 inference system- (MFIS-) based expert system (BCP-T1F) is explained in this section. The BCP-T1F expert system consists of four layers as shown in Figure 2. In layer 1 named symptoms, the initial symptoms of breast cancer will be checked which are swelling, breast pain, redness, nipple retraction, Family Inheritance Breast Cancer (FBIC), and skin irritation. This will find whether it is lump or cancer.

If the system finds the symptoms in the patient, then layer 2 diagnoses the breast cancer (no/yes) using two input variables that are ultrasound and mammography. If the layer 2 diagnoses breast cancer, then layer 3 will be activated. Layer 3 predicts the type and severity of BCP based on two input variables (biopsy gold severity) and (biopsy gold type). Then layer 4 will check the stage of cancer by three input variables that are MRI, CT, and PET which are shown in Figure 2.

Mathematically, the layers of the proposed BCP-T1F can be written as follows.

This layer 1 can be written mathematically as

(1) $$\begin{array}{c} \mu_{\text{DBI},\text{Layer}1}=\text{MFIS}\left[\mu_{\text{swelling}},\mu_{\text{Breast pain}},\mu_{\text{Redness}},\mu_{\text{Skin irritation}},\mu_{\text{FBIC}},\mu_{\text{nipple}-\text{retraction}}\right]. \end{array}$$

The layer 2 can be written as

(2) $$\begin{array}{c} \mu_{\text{DBI}-\text{RADS},\text{Layer }2}=\text{MFIS}\left[\mu_{\text{Ultrasond}},\mu_{\text{Mammography}}\right]. \end{array}$$

Then layer 3 can be written as

(3) $$\begin{array}{c} \mu_{\text{DBC}-\text{ST},\text{ Layer }3}=\text{MFIS}\left[\mu_{\text{Biopsy Gold}-\text{Type}},\mu_{\text{Biopsy Gold}-\text{Severity}}\right]. \end{array}$$

Then layer 4 can be written as

(4) $$\begin{array}{c} \mu_{\text{DBC}-\text{ST},\text{ Layer }4}=\text{MFIS}\left[\mu_{\text{MRI}},\mu_{\text{CT}},\mu_{\text{PET}}\right]. \end{array}$$

#### 3.1.1. Membership Functions

The membership function of proposed BCP-T1F expert system yields the curve values ranging between 0 and 1 and also dispenses a mathematical form of the fuzzy logic that accords statistical values of both the input and output variables. The mathematical representation of proposed BCP-T1F expert system yields member functions of layers 1–4 shown in Tables 1–4. These membership functions are gathered after the consultation with the medical experts from Cavan General Hospital Lisdaran, Cavan, Ireland.

#### 3.1.2. Rules Table

The proposed system BCP-T1F rules table usually relies on the expert system which constitutes of sixty-four inputs and output rules for layer 1, fifteen output and input rules for layer 2, eight input and output rules for layer 3, and thirty I/O rules for layer 4. This rule (Table 5) has been obtained with the assistance of the medical experts from Cavan General Hospital Lisdaran, Cavan, Ireland.

#### 3.1.3. Rule Based

Rules are essential for input and output variables. Achievement of an adroit system is built based on rules. Some of the rules are shown in Table 4.

#### 3.1.4. Inference Engine

Inference engine is the most emphasized constituent of any decision-based expert system. In this manuscript, BCP-T1F expert system has been employed in layer 1, layer 2, layer 3, and layer 4.

#### 3.1.5. Defuzzification

**Figure 3:**
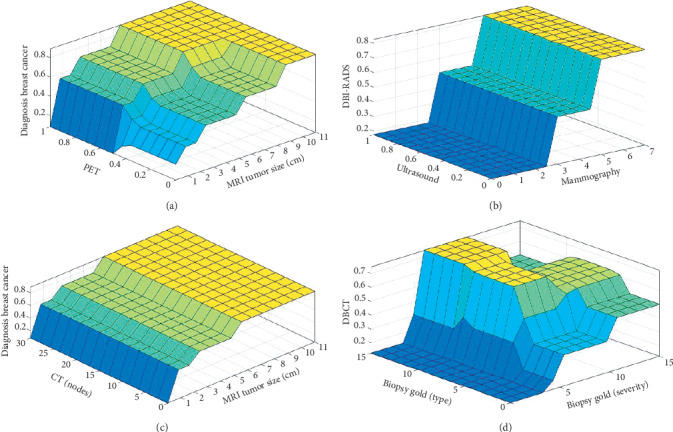
(a) Rule surface for PET and MRI tumor size. (b) Rule surface for ultrasound and mammography. (c) Rule surface for CT (nodes) and MRI tumor size. (d) Rule surface for biopsy gold (type) and biopsy gold (severity).

Defuzzification is the process of making a measureable result in crusty logic, given fuzzy sets, and corresponding membership degrees. It is the process that plots a fuzzy set to a crisp set. It is characteristically needed in fuzzy control systems. In Figures 3(a)–3(d), the graphical illustrations of defuzzifier of BCP-T1F expert system are obtainable.

#### 3.1.6. Lookup Diagram

**Figure 4:**
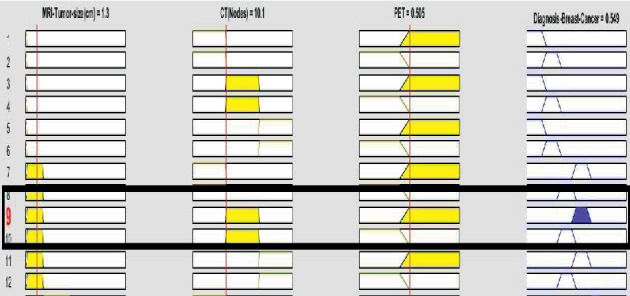
Lookup diagram for proposed BCP-T1F expert system.

MATLAB R2019a tool is used for demonstrating, imitation, algorithm expansion, prototyping, and various other fields. This tool is well organized for software designing, data examination, conception, and calculations. For the simulation of results, three inputs and one output of BCP are used on layer 4 which are shown in Figure 4.

Figure 4 shows that (*μ*_CT nodes mode_(*o*)) is considered as node 2, (*μ*_MRI−tumor size_(*ν*)) is taken into account as low size, (*μ*_PET_(*θ*)) is spread in whole body, and (*μ*_DBC−stage_(*χ*)) turns out to be concluded as Stage 3.

Similarly, Figure 4 also demonstrates the rule-based knowledge; few of them are shown as follows:

(*μ*_CT nodes mode_(*o*)) is considered as node 3, (*μ*_MRI−tumor size_(*ν*)) is taken into account as very high size, (*μ*_PET−benign_(*θ*)) is spread in whole body, and (*μ*_DBC−stage _(*χ*)) turns out to be concluded as Stage 4.

(*μ*_CT nodes mode_(*o*)) is considered as node 2, (*μ*_MRI−tumor size_(*ν*)) is taken into account as low size, (*μ*_PET−benign_(*θ*)) is spread in whole body, and (*μ*_DBC−stage _(*χ*)) turns out to be concluded as Stage 2.

(*μ*_CT nodes mode_(*o*)) is considered as node 1, (*μ*_MRI−tumor size_(*ν*)) is taken into account as no tumor, (*μ*_PET−benign_(*θ*)) is benign, and (*μ*_DBC−stage_(*χ*)) turns out to be concluded as Stage 0.

(*μ*_CT nodes mode_(*o*)) is considered as node 2, (*μ*_MRI−tumor size_(*ν*)) is taken into account as low size, (*μ*_PET−benign_(*θ*)) is benign, and (*μ*_DBC−stage_(*χ*)) turns out to be concluded as Stage 1.

### 3.2. SVM-Based System Model

#### 3.2.1. Sensor Data

Heterogeneous sensors are collecting continuously environmental data. It is transforming a physical quantity into a measurement. Multiple sensors are connected in the form of topology with the sensor board. Each sensor node acquires a subset of the collected samples for locally compressing and summarizing from the random signal.

#### 3.2.2. Preprocessing

Data preprocessing is a data mining technique collecting the data from the patients which involves transforming raw data into an understandable format. Real-world data is often incomplete, is inconsistent, and is likely to contain many errors. In this step, we handle the missing values using mean, mode, and so on. We also mitigate the noisy data using the moving average method in which we used five-filter size. Data preprocessing prepares raw data for further processing.

**Figure 5:**
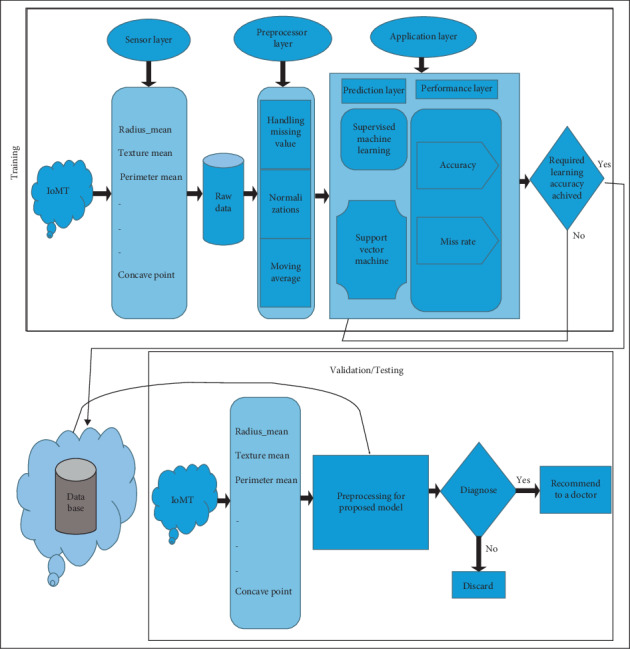
Proposed BCP-SVM expert system methodology.

In this article, Figure 5 has proposed a new system model for breast cancer control using support vector machine system in ML [48] and for breast cancer prediction BCP-SVM. This model depicts the whole process through picturing of the proposed BCP-SVM system model. With the help of this model, we can witness that the data gained from the Internet of medical things is utilized in sensory layer. This fed data can be updated with the help sensors. The layer named sensory layer has all the parameters which will be employed to predict cancer. The outcome generated is in the form of raw data. The raw data will be fed into the preprocessing layer. Data preprocessing prepares raw data for further processing. The raw data goes through the managing, moving, and normalization in the preprocessing layer. The portable standard was employed to eliminate inconsistencies from the data which is done in the previous layers termed as a preprocessing layer. After the data from preprocessing layer jumps on to the application layer, this layer of various parameters which are used in the application layer finds out the breast cancer malignancy. The layer is divided into two halves known as performance layer and prediction layer. In the prediction layer, Support Vector Machine is used to find out the breast cancer, and in the performance layer, it is used to evaluate the results produced by the prediction layer performance which are shown in Figure 5. The application layer evaluates the data being fed into this layer which then gives out whether the accuracy is achieved or not.

The proposed model is categorized into five different layers. If the trained accuracy is achieved, then it is passed onto the cloud for the further proceeding for the validation process. Cloud stores the data whether it is for training process or for testing process.

From cloud, data is being received for the validation process. The trained data or input is fed into the cloud to determine an evaluation system for the testing purposes. It is fed into the cloud and then forwarded to the preprocessing layer [51] where data is improved by handling missing values and errors; then finally it is transferred to the further diagnosis.

As we know, the equation of the line is

(5) $$\begin{array}{c} j_{2}=xj_{1}+y, \end{array}$$

where “*x*” is slope of a line and “*y*” is the intersect; therefore,

(6) $$\begin{array}{c} xj_{1}-j_{2}+y=0. \end{array}$$

Let $\overset{⟶}{j\;}={\left(j_{1},j_{2}\right)}^{T}$ and $\overset{⟶}{i\;}=\left(x,-1\right)$; then the above equation can be written as

(7) $$\begin{array}{c} \overset{⟶}{i}\cdot \overset{⟶}{j}=0. \end{array}$$

This equation is obtained from 2-dimensional vectors. It also works for different number of dimensions; (6) depicts the general equation of hyperlane.

The direction of a vector $\overset{⟶}{j}={\left(j_{1},j_{2}\right)}^{T}$ is written as $\overset{⟶}{i}$ and is defined as

(8) $$\begin{array}{c} i=\frac{j_{1}}{\left\|j\right\|}+\frac{j_{2}}{\left\|j\right\|}, \end{array}$$

where

(9) $$\begin{array}{c} \left\|j\right\|=\sqrt{j_{1+\;}^{2}j_{2+\;}^{2}j_{3+\;}^{2}\ldots j_{n\;}^{2}}. \end{array}$$

As we know,

(10) $$\begin{array}{c} \cos\left(\theta \right)=\frac{j_{1}}{\left\|j\right\|},\\ \cos\left(\gamma \right)=\frac{j_{2}}{\left\|j\right\|}. \end{array}$$

Equation (3) can also be written as

(11) $$\begin{array}{c} w=\left(\cos\left(\theta \right),\;\cos\left(\gamma \right)\right),\\ \overset{⟶}{i\;}\cdot \overset{⟶}{j}=\left\|i\right\|\left\|j\right\|\cos\left(\theta \right),\\ \theta =\;\eta -\;\gamma \;\cos\left(\theta \right)=\cos\left(\eta -\gamma \right)\\ =\cos\left(\eta \right)\cos\left(\gamma \right)+\sin\left(\eta \right)\sin\left(\gamma \right)\\ =\frac{i_{1}}{\left\|i\right\|}\frac{j_{1}}{\left\|j\right\|}+\;\frac{i_{2}}{\left\|i\right\|}\;\frac{j_{2}}{\left\|j\right\|}\;\\ =\;\frac{i_{1}j_{1}+\;i_{2}j_{2}}{\left\|j\right\|\left\|j\right\|},\\ i\cdot j=\left\|i\right\|\left\|j\right\|\left[\frac{i_{1}j_{1}+\;i_{2}j_{2}}{\left\|i\right\|\left\|j\right\|}\right],\\ i\cdot j=\sum_{k=1}^{n}i_{k}j_{k}. \end{array}$$

The dot product can be computed as the above equation for *n*-dimensional vectors.

Let

(12) $$\begin{array}{c} z=m\left(i\cdot j+y\right). \end{array}$$

If sign (*z*) > 0, then it is correctly classified, and if sign (*z*) < 0, then it is incorrectly classified.

Given a dataset *D*, we compute *f* on a training dataset:

(13) $$\begin{array}{c} z_{k}=m_{k}\left(i\cdot j+y\right). \end{array}$$

Then *Z* which is called functional margin of the dataset is as follows:

(14) $$\begin{array}{c} Z=\underset{k=1.\ldots .n}{\min}z_{k}. \end{array}$$

Taking hyperplanes, the hyperplane with the largest *Z* will be commendatory selected. The geometric margin of the dataset is denoted by *Z*. The main goal is to take into account an optimal hyperplane, which means finding the values of $\overset{⟶}{i\;}$ and *y* of the optimal hyperplane.

The Lagrangian function shows the following equation:

(15) $$\begin{array}{c} \psi \left(i,y,x\right)=\frac{1}{2}i\cdot i-\sum_{k=1}^{n}\gamma_{k}\left[m:\left(i\cdot j+y\right)-1\right], \end{array}$$

(16) $$\begin{array}{c} \phi_{i}\psi \left(i,y,x\right)=i-\sum_{k=1}^{n}\gamma_{k}m_{k}j_{k}=0, \end{array}$$

(17) $$\begin{array}{c} \phi_{y}\psi \left(i,y,x\right)=-\sum_{k=1}^{n}\gamma_{k}m_{k}=0. \end{array}$$

From (16) and (17), we get

(18) $$\begin{array}{c} i=\sum_{k=1}^{n}\gamma_{k}m_{k}j_{k},\\ \sum_{k=1}^{n}\gamma_{k}m_{k}=0. \end{array}$$

After substituting the Lagrangian function *ψ*, we get

(19) $$\begin{array}{c} i\left(\gamma ,y\right)=\sum_{k=1}^{n}\gamma_{k}-\frac{1}{2}\sum_{k=1}^{n}\sum_{l=1}^{n}\gamma_{k}\gamma_{k}m_{k}m_{l}j_{k}j_{l}, \end{array}$$

and thus

(20) $$\begin{array}{c} \;\begin{array}{cc} \underset{\gamma }{\max} & \sum_{k=1}^{n}\gamma_{i}-\frac{1}{2}\sum_{k=1}^{n}\sum_{l=1}^{n}\gamma_{k}\gamma_{l}m_{k}m_{l}j_{k}j_{l}\\ \text{Subject to} & \gamma_{k}\;\geq 0,\;k=1,\ldots ,n,\;\sum_{k=1}^{n}\gamma_{k}m_{k}=0. \end{array} \end{array}$$

The expansion of the Lagrangian multipliers method to the Karush–Kuhn–Tucker conditions can be done; the constraints will bear disproportion. The Karush–Kuhn–Tucker commendatory conditions will be expressed as

(21) $$\begin{array}{c} \gamma_{k}\left[m_{k}\left(i_{k}\cdot j^{*}+y\right)-1\right]=0, \end{array}$$

where *j*^*∗*^ is the optimal point, *γ* is the positive value, and *α* for the other points are ≈0.

So,

(22) $$\begin{array}{c} m_{k}\left(\left(i_{k}\cdot j^{*}+y\right)-1\right)=0. \end{array}$$

These are called support vectors, which are the closest points to the hyperplane. According to (22),

(23) $$\begin{array}{c} i-\sum_{k=1}^{n}\gamma_{k}m_{k}j_{k}=0,\\ i=\sum_{k=1}^{n}\gamma_{k}m_{k}j_{k}. \end{array}$$

To compute the value of *y*, we get

(24) $$\begin{array}{c} m_{k}\left(\left(i_{k}\cdot j^{*}+y\right)-1\right)=0. \end{array}$$

Multiplying both sides by *m* in (24), then we get

(25) $$\begin{array}{c} m_{k}^{2}\left(\left(j_{k}\cdot j^{*}+y\right)-m_{k}\right)=0, \end{array}$$

where *m*_*k*_^2^=1;

(26) $$\begin{array}{c} \left(\left(i_{k}\cdot j^{*}+y\right)-m_{k}\right)=0,\\ y=m_{k}-i_{k}\cdot j^{*}. \end{array}$$

Then

(27) $$\begin{array}{c} y=\frac{1}{S}\sum_{k=1}^{s}\left(m_{k}-i_{k}\cdot j\right). \end{array}$$

The number of support vectors is *S*; we will have the hyperplane. To make predictions, hyperplane is used. And the hypothesis function is as follows:

(28) $$\begin{array}{c} h\left(i_{k}\right)=\left[\begin{array}{cc} +1 & \text{if }i\cdot j+y\geq 0\\ -1 & \text{if }i\cdot j+y<0 \end{array}\right]. \end{array}$$

The above point which arises on the hyperplane will be considered as class +1 (breast cancer found) and the point which lies down the hyperplane will be categorized as −1 (breast cancer not found).

So, basically, the objective of the SM algorithm is to find a hyperplane which could separate the data accurately and we need to find the best one, which is often referred to as the optimal hyperplane.

## 4. Simulation and Results

MATLAB 2019a is used for simulation purpose.  Section 4.1 contains the results of proposed fuzzy-based model and Section 4.2 contains the result of proposed SVM-based model.

### 4.1. Fuzzy Results

**Figure 6:**
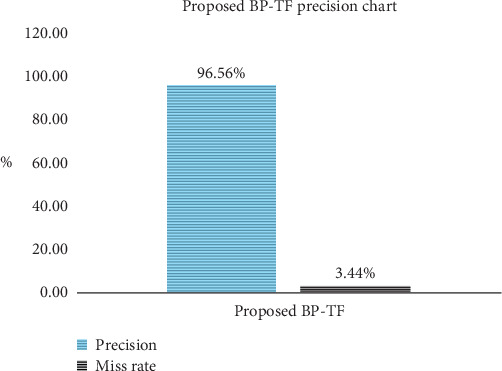
Precision chart of proposed BCP-T1F.

For the constructive results, MATLAB R2019a is used as a tool so as to gather the stimulation of results taking algorithm development along with it; it also takes prototyping into account. The interpretation of the results is being developed by taking the 12 total inputs and 4 outputs variables for fuzzy logic. When layer 1 shows the symptoms to be found in a particular person, then it rushes to the second layer in which mammography and ultrasound are done to do the initial treatment so as to assure that something is fishy going on; in this research, the proposed BCP-T1F system not only diagnoses the disease but also shows the different levels. When jumping towards layer 3, it depicts that the biopsy gold determines the type and severity of the lump, whether it is cancerous or not. Then the third layer comes; it involves PET, MRI, and CT which in turns gives the size of the tumor and to which extent it is cancerous.  Figure 6 has clearly stated the precision of a proposed system. The proposed system BCP-T1F shows the precision rate of 96.56 percent and the miss rate of the BCP-T1F comes out to be 3.44 percent. This proposed system is providing the accurate results for the corresponding type and severity level.

### 4.2. SVM Results

The simulation of MATLAB R2019a tool is employed to assume and predict the breast cancer. Tables 6 and 7 conclude the training and validation with respect to precision rate and miss rate. SVM algorithm has been implemented to the dataset [15] of 569 sets of records; moreover, this data is divided into training constitutes of 70% (399 samples) and 30% (170 samples) for the mentioned purposes training and validation. Various statistical measures used for comparing as well as performance are calculated with different metrics named sensitivity, specificity, and accuracy, whereas the true positivity is expressed in sensitivity and accurate negative as specificity. The following parameters are derived by the formulas given as follows:

(29) $$\begin{array}{c} \text{Misrate}=\frac{\left(O_{M}/E_{B}\right)+\left(O_{B}/E_{M}\right)}{E_{B}+E_{M}},\\ \text{Precision}=\frac{\left(O_{B}/E_{B}\right)+\left(O_{M}/E_{M}\right)}{E_{B}+E_{M}},\\ \text{Sensitivity}=\frac{\left(O_{M}/E_{M}\right)}{\left(O_{M}/E_{M}\right)+\left(O_{B}/E_{B}\right)},\\ \text{Specificity}=\frac{\left(O_{B}/E_{B}\right)}{\left(\left(O_{M}/E_{M}\right)+\left(O_{B}/E_{B}\right)\right)},\\ \text{Negative prediction value}=\frac{\left(O_{B}/E_{B}\right)}{\left(\left(O_{M}/E_{B}\right)+\left(O_{B}/E_{E}\right)\right)},\\ \text{Positive prediction value}=\frac{\left(O_{M}/E_{M}\right)}{\left(\left(O_{M}/E_{M}\right)+\left(O_{B}/E_{M}\right)\right)},\\ \text{False positive ratio }=1-\text{specificity,}\\ \text{False negative ratio }=1-\text{sensitivity,}\\ \text{Likelihood ratio positive}=\frac{\text{sensitivity}}{\left(1-\text{specificity}\right)},\\ \text{Likelihood ratio negative}=\frac{\left(1-\text{sensitivity}\right)}{\text{specificity}}. \end{array}$$

The proposed BCP-SVM system model calculates the predicted output as negative (−1) and positive (1). The resultant output of value negative (−1) shows that there is benign and positive (1) value which shows the existence of malignancy.

Table 6 shows the proposed BCP-SVM system model prediction of breast cancer during the training phase. Total 399 number of samples are used during training which is further divided into 250,149 samples of positive and negative, respectively. It is observed that 248 samples have positive class which are correctly predicted and no breast cancer (benign) is found but 02 records are incorrectly predicted as a negative which means breast cancer (malignancy) is found. Similarly, total 149 samples are taken, wherein the case of negative means congestion is found, in which 144 samples are correctly predicted as a negative which means breast cancer is found and 05 samples are invalidly predicted as a positive which means no breast cancer is found, while actually breast cancer exists there.

Table 7 shows the proposed BCP-SVM system model prediction of breast cancer during validation phase. Total 170 numbers of samples are used during training which further are divided into 107,63 samples of positive and negative, respectively. It is observed that 106 samples of positive class have no breast cancer found and also are correctly predicted but 01 records are incorrectly predicted as a negative which means breast cancer is found, while actually breast cancer does not exist. Similarly, total 63 samples are taken in the case of negative which means breast cancer is found, in which 59 samples are correctly predicted as a negative which means breast cancer is found and 04 samples are invalidly predicted as a positive which means no breast cancer is found, while actually breast cancer existed there.

Table 8 shows the proposed BCP-T1F-SVM system model performance in terms of sensitivity, specificity, precision, and miss rate during training and testing phase. It clearly shows that the proposed BCP-T1F-SVM system during training gives 98.63%, 98.02%, 98.25%, and 1.75% sensitivity, specificity, accuracy, and miss rate, respectively. And during testing, the proposed BCP-T1F-SVM system gives 98.33%, 96.36%, 97.06%, and 2.94% sensitivity, specificity, accuracy, and miss rate, respectively. In addition, some more statistical measures are added to predict the values such as false positive, false negative, likelihood ratio negative, and positive and positive and negative prediction values give the result during training 1.98%, 1.37%, 0.0139, 49.81, 96.64%, and 99.2%. And during testing, the proposed TCC-SVM system gives 3.64%, 1.67%, 0.0173, 27.01, 93.65%, and 99.06%, respectively.

**Figure 7:**
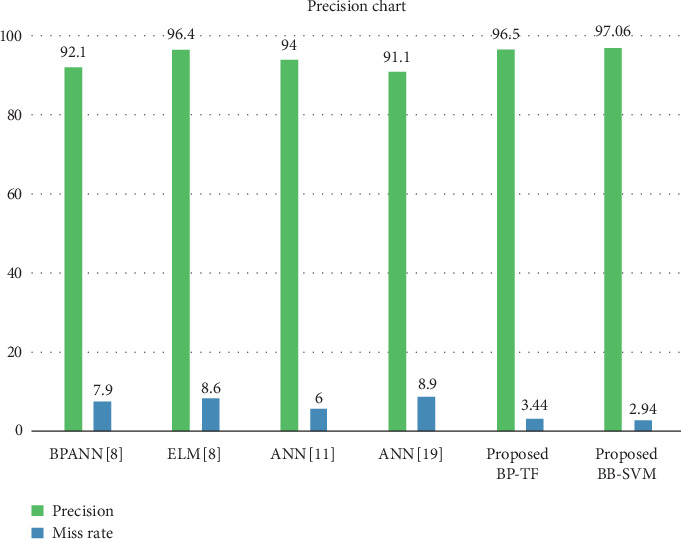
Comparisons with previous methods.

Table 9 and Figure 7 show the performance of the proposed BCP-T1F-SVM system model using fuzzy logic and SVM with previous approaches given in the literature BCP-ANN [8], ANN-ELM [8], and ANN [11, 17].

## 5. Conclusion

For the constructive results, MATLAB R2019a is used as a tool so as to gather the stimulation of results taking algorithm development along with it; it also takes prototyping into account. The interpretation of the results is being developed by taking the 12 total inputs and 4 outputs variables for fuzzy logic and 30 inputs and 1 output variables for SVM. The main goal is to analyze critically the different dimensions of breast cancer or any type of cancerous disease. The proposed system BCP-T1F-SVM is to devise a type of expert system to diagnose breast cancer and its stages. The reports on the basis of which the expert system analysis has been carried out were yielded by Cavan General Hospital Lisdaran, Cavan, Ireland. This expert system can be employed by the medical specialists and nonspecialist also. In this article, the proposed principal expert system BCP-T1F achieved a precision of 96.56 percent, which in turn also accords the 3.44 percent of miss rate. The proposed BCP-SVM is proven to provide an accuracy of 97.06 percent which in turn also accords the 2.94 percent of miss rate. Both the proposed BCP-T1F and BCP-SVM systems give more accuracy as compared to a previous published approach.

## Figures and Tables

**Table 1:** Input and output variables membership functions used in the proposed BCP-T1F expert system.

Sr. number	I/P parameters	Mathematics of membership function
1	Swelling(*μ*_Swelling_(*α*))	*μ*_Swelling−affected_(*α*)={max(min(1, ((0.5 − *α*)/0.1)), 0)}, *μ*_Swelling−not affected_(*α*)={max(min(1, ((*α* − 0.4)/0.1) ), 0)}

2	Skin irritation(*μ*_Skin−irritation_(*β*))	*μ*_Skin−irritation,affected_(*β*)={max(min(1, ((1.5 − *β*)/0.1)), 0)}, *μ*_Skin−irritation,not affected_(*β*)={max(min(1, ((*β* − 1.4)/0.1)), 0)}

3	Breast pain (*μ*_Breast−pain_(*γ*))	*μ*_Breast pain−affected_(*γ*)={max(min(1, ((2.5 − *γ*)/0.1)), 0)}, *μ*_Breast pain−not affected_(*γ*)={max(min(1, ((*γ* − 2.4)/0.1)), 0)}

4	Redness(*μ*_Redness_(*ρ*))	*μ*_Redness−affected_(*ρ*)={max(min(1, ((3.5 − *ρ*)/0.1)), 0)}, *μ*_Redness−not affected_(*ρ*)={max(min(1, ((*ρ* − 3.4)/0.1)), 0)}

5	Family inheritance, Breast cancer(*μ*_FIBC_(*ψ*))	*μ*_*E*,*N*_(*ψ*)={max(min(1, ((4.5 − *ψ*)/0.1)), 0)}, *μ*_*E*,*P*_(*ψ*)={max(min(1, ((*ψ* − 4.4)/0.1)), 0)}

6	Nipple retraction (*μ*_Nipple−retraction_(*φ*))	*μ*_Nipple retraction−affected_(*φ*)={max(min(1, ((5.5 − *φ*)/0.1)), 0)}, *μ*_Nipple retraction−not affected_(*φ*)={max(min(1, ((*φ* − 5.4)/0.1)), 0)}

7	Diagnosis infection (*μ*_DI_(*di*))	$\mu_{\text{DI}-\text{normal}}\left(di\right)=\left\{\begin{array}{cc} 1, & \text{if }di\;€\left[\begin{array}{cc} 0 & 0.4 \end{array}\right],\\ \left(\left(0.5-di\right)/0.1\right), & \text{if }di\;€\left[\begin{array}{cc} 0.4 & 0.5 \end{array}\right],\\ 0, & \text{otherwise} \end{array}\right\}$, $\mu_{\text{DI}-\text{infected}}\left(di\right)=\;\left\{\begin{array}{cc} \left(\left(di-0.4\right)/0.1\right), & \text{if }di\;€\left[\begin{array}{cc} 0.4 & 0.5 \end{array}\right]\\ 1, & \text{if }di\;€\left[\begin{array}{cc} 0.5 & 1 \end{array}\right]\\ 0, & \text{otherwise} \end{array}\right\}$

**Table 2:** Input and output variables membership functions used in the proposed BCP-T1F expert system.

Sr. number	I/P parameters	Mathematics of membership function
1	Ultrasound (*μ*_Ultrasound_(*λ*))	*μ*_Swelling−affected_(*λ*)={max(min(1, ((0.5 − *λ*)/0.1)), 0)}, *μ*_Swelling−not affected_(*λ*)={max(min(1, ((*λ* − 0.4)/0.1)), 0)}

2	Mammography (*μ*_Mammography_(*τ*))	*μ*_Mammography−incomplete_(*τ*_1_)={max(min(1, ((0.9 − *τ*_1_)/0.1)), 0)}, *μ*_Mammography−negative_(*τ*_2_)={max(min(((*τ*_2_ − 0.8)/0.1), 1, ((1.9 − *τ*_2_)/0.1)))}, *μ*_Mammography−benign_(*τ*_3_)={max(min(((*τ*_3_ − 1.8)/0.1), 1, ((2.9 − *τ*_3_)/0.1)))}, *μ*_Mammography−probably benign_(*τ*_4_)={max(min(((*τ*_4_ − 2.8)/0.1), 1, ((3.9 − *τ*_4_)/0.1)))}, *μ*_Mammography−suspicious_(*τ*_5_)={max(min(((*τ*_5_ − 3.8)/0.1), 1, ((4.9 − *τ*_5_)/0.1) ))}, *μ*_Mammography−suggested malignancy_(*τ*_6_)={max(min(((*τ*_6_ − 4.8)/0.1), 1, ((5.9 − *τ*_6_)/0.1)))}, ${\begin{array}{c} \mu \end{array}}_{\text{Mammography}-\text{proven malignnancy}}\left(\tau_{7}\right)=\left\{\max\left(\min\left(1,\left(\left(\tau_{7}-5.8\right)/0.1\right),0\right)\right)\right\}$

3	Detection of breast imaging reporting and database system score (*μ*_DBI−rads_(rads))	$\mu_{\text{DBI}-\text{Rads}-\text{No lump}}\left(\text{rads}\right)=\left\{\begin{array}{cc} 1, & \text{if rads}€\left[\begin{array}{cc} 0 & 0.3 \end{array}\right]\\ \left(\left(0.4-\text{rads}\right)/0.1\right) & \text{if rads}€\left[\begin{array}{cc} 0.3 & 0.4 \end{array}\right]\\ \text{0,} & \text{otherwise} \end{array}\right\}$, $\mu_{\text{DBI}-\text{Rads}-\text{Lump}}\left(\text{rads}\right)=\left\{\begin{array}{cc} \left(\left(\text{rads}-0.3\right)/0.1\right), & \text{if rads}€\left[\begin{array}{cc} 0.3 & 0.4 \end{array}\right]\\ 1, & \text{if rads}€\left[\begin{array}{cc} 0.4 & 0.6 \end{array}\right]\\ \left(\left(0.7-\text{rads}\right)/0.1\right) & \text{if rads}\left[\begin{array}{cc} 0.6 & 0.7 \end{array}\right]\\ 0, & \text{otherwise} \end{array}\right\}$, $\mu_{\text{DBI}-\text{Rads}-\text{Cancerous lump}}\left(\text{rads}\right)=\left\{\begin{array}{cc} 1, & \text{if rads}€\left[\begin{array}{cc} 0.7 & 1 \end{array}\right]\\ \left(\left(\text{rads}-0.6\right)/0.1\right) & \text{if rads}€\left[\begin{array}{cc} 0.6 & 0.7 \end{array}\right]\\ 0, & \text{otherwise} \end{array}\right\}$

**Table 3:** Input and output variables membership functions used in the proposed BCP-T1F expert system.

Sr. number	I/P parameters	Mathematics of membership function
1	Biopsy gold standard for severity (*μ*_Biopsy gold−severity_(*δ*))	*μ*_Biopsy gold−severity−benign_ (*δ*_1_)={max(min(1, ((5 − *δ*_1_)/1)), 0)}, *μ*_Biopsy gold−severity−cancer−severity low_(*δ*_2_)={max(min(((*δ*_2_ − 4)/1), 1, ((10 − *δ*_2_)/1)), 0)}, *μ*_Biopsy gold−severity−cancer−severity high_(*δ*_3_)={max(min(1, ((*δ*_3_ − 9)/1)), 0)}

2	Biopsy gold standard for type (*μ*_Biopsy gold−type_(*π*))	*μ*_Biopsy gold−type−fine needle aspiration_(*π*_1_)={max(min(1, ((5 − *π*_1_)/1)), 0)}, *μ*_Biopsy *g*old−type−core needle biopsy_(*π*_2_)={max(min(((*π*_2_ − 4)/1), 1, ((10 − *π*_2_)/1)), 0)}, *μ*_Biopsy gold−type−surgical biopsy_(*π*_3_)={max(min(1, ((*π*_3_ − 9)/1)), 0)}

3	Diagnosis of breast cancer severity (*μ*_DBCS_(*ρ*))	$\mu_{\text{DBCS}-\text{mild grade}}\left(\rho_{1}\right)=\left\{\begin{array}{cc} 1, & \text{if }\in \left[\begin{array}{cc} 0 & 0.3 \end{array}\right]\\ \left(\left(0.4-\rho_{1}\right)/0.1\right), & \text{if }\in \left[\begin{array}{cc} 0.3 & 0.4 \end{array}\right]\\ 0, & \text{otherwise} \end{array}\right\}$, $\mu_{\text{DBCS}-\text{moderate severity}}\left(\rho_{2}\right)=\left\{\begin{array}{cc} \left(\left(\rho_{2}-0.3\right)/0.1\right), & \text{if}\in \left[\begin{array}{cc} 0.3 & 0.4 \end{array}\right]\\ 1, & \text{if}\in \left[\begin{array}{cc} 0.4 & 0.6 \end{array}\right]\\ \left(\left(0.7-\rho_{2}\right)/0.1\right), & \text{if}\in \left[\begin{array}{cc} 0.6 & 0.7 \end{array}\right]\\ 0, & \text{otherwise} \end{array}\right\}$, $\mu_{\text{DBCS}-\text{high severity}}\left(\rho_{3}\right)=\left\{\begin{array}{cc} 1, & \text{if rads}\in \left[\begin{array}{cc} \text{0.}7 & 1 \end{array}\right]\\ \left(\left(\rho_{3}-0.6\right)/0.1\right), & \text{if rads}\in \left[\begin{array}{cc} \text{0.}6 & 0.7 \end{array}\right]\\ 0, & \text{otherwise} \end{array}\right\}$

4	Diagnosis of breast cancer type (*μ*_DBCT_(*б*))	$\mu_{\text{DBCT}-\text{severity}}\left({б}_{1}\right)=\left\{\begin{array}{cc} 1, & \text{if}\in \left[\begin{array}{cc} \text{0} & 0.2 \end{array}\right]\\ \left(\left(0.3-{б}_{1}\right)/0.1\right) & \text{if}\in \left[\begin{array}{cc} \text{0.}2 & 0.3 \end{array}\right]\\ \text{0,} & \text{otherwise} \end{array}\right\}$, $\mu_{\text{DBCT}-\text{ductal carcinoma}}\left({б}_{2}\right)=\left\{\begin{array}{cc} \left(\left({б}_{2}-0.2\right)/0.1\right), & \text{if}\in \left[\begin{array}{cc} \text{0.}2 & 0.3 \end{array}\right]\\ 1, & \text{if}\in \left[\begin{array}{cc} \text{0.}3 & 0.4 \end{array}\right]\\ \left(\left(0.5-{б}_{2}\right)/0.1\right) & \text{if}\in \left[\begin{array}{cc} \text{0.}4 & 0.5 \end{array}\right]\\ 0, & \text{otherwise} \end{array}\right\}$, $\mu_{\text{DBCT}-\text{invasive lobular carcinoma}}\left({б}_{3}\right)=\left\{\begin{array}{cc} 1, & \text{if}\in \left[\begin{array}{cc} 0.5 & 0.6 \end{array}\right]\\ \left(\left({б}_{3}-0.4\right)/0.1\right), & \text{if}\in \left[\begin{array}{cc} 0.4 & 0.5 \end{array}\right]\\ \left(\left(0.7-{б}_{3}\right)/0.1\right), & \text{if}\in \left[\begin{array}{cc} 0.6 & 0.7 \end{array}\right]\\ 0, & \text{otherwise} \end{array}\right\}$, $\mu_{\text{DBCT}-\text{inflammatory breast disease}}\left({б}_{4}\right)=\left\{\begin{array}{cc} 1, & \text{if}\in \left[\begin{array}{cc} 0.6 & 0.7 \end{array}\right]\\ \left(\left({б}_{4}-0.6\right)/0.1\right), & \text{if}\in \left[\begin{array}{cc} 0.7 & 0.8 \end{array}\right]\\ \left(\left(0.9-{б}_{4}\right)/0.1\right), & \text{if}\in \left[\begin{array}{cc} 0.8 & 0.9 \end{array}\right]\\ 0, & \text{otherwise} \end{array}\right\}$, $\;\mu_{\text{DBCT}-\text{lobular carcinoma}}\left({б}_{5}\right)=\left\{\begin{array}{cc} 1, & \text{if}\in \left[\begin{array}{cc} \text{0.}9 & 1 \end{array}\right]\\ \left(\left({б}_{5}-0.8\right)/0.1\right), & \text{if}\in \left[\begin{array}{cc} \text{0.}8 & 0.9 \end{array}\right]\\ 0, & \text{otherwise} \end{array}\right\}$

**Table 4:** Input and output variables membership functions used in the proposed BCP-T1F expert system.

Sr. number	I/P parameters	Mathematics of membership function
1	Computed tomography (CT) (*μ*_CT nodes mode_(*o*))	$\left(\mu_{\text{CT nodes }-\text{ mode }1}\left(o\right)\right)=\left\{\begin{array}{cc} 1, & \text{if }o\in \left[\begin{array}{cc} 0 & 7.9 \end{array}\right]\\ \left(\left(10-o\right)|/|\left(10-9.9\right)\right), & \text{if }o\in \left[\begin{array}{cc} 9.9 & 10 \end{array}\right]\\ 0, & \text{otherwise} \end{array}\right\}$, $\left(\mu_{\text{CT nodes }-\text{ mode }1}\left({ο}_{2}\right)\right)=\left\{\begin{array}{cc} \left(\left(o-9.9\right)|/|\left(10-9.9\right)\right), & \text{if }o\in \left[\begin{array}{cc} 9.9 & 10 \end{array}\right]\\ 1, & \text{if }o\in \left[\begin{array}{cc} 10 & 19.9 \end{array}\right]\\ \left(\left(20-o\right)|/|\left(20-9.9\right)\right), & \text{if }o\in \left[\begin{array}{cc} 19.9 & 20 \end{array}\right]\\ 0, & \text{otherwise} \end{array}\right\}$, $\left(\mu_{\text{CT nodes }-\text{ mode }1}\left({ο}_{3}\right)\right)=\left\{\begin{array}{cc} \left(\left(o-19.9\right)|/|\left(20-19.9\right)\right), & \text{if }o\in \left[\begin{array}{cc} 19\text{.}9 & 20 \end{array}\right]\\ 1, & \text{if }o\in \left[\begin{array}{cc} 20 & 30 \end{array}\right]\\ \left(\left(20-o\right)|/|\left(20-9.9\right)\right), & \text{if }o\in \left[\begin{array}{cc} 19\text{.}9 & 20 \end{array}\right]\\ 0, & \text{otherwise} \end{array}\right\}$

2	Magnetic resonance imaging (*μ*_MRI−tumor *s*ize_(*ν*))	$\mu_{\text{MRI }-\text{ tumor size}-\text{ no}}\left(\nu \right)=\left\{\begin{array}{cc} 1, & \text{if }\nu \in \left[\begin{array}{cc} 0.02 & 0.1 \end{array}\right]\\ \left(\left(0.2-\nu \right)/0.1\right) & \text{if }\nu \in \left[\begin{array}{cc} 0.1 & 0.2 \end{array}\right]\\ 0, & \text{otherwise} \end{array}\right\}$, $\mu_{\text{MRI}-\text{tumor size}-\text{low}}\left(\nu \right)=\left\{\begin{array}{cc} \left(\left(\nu -0.1\right)/0.1\right), & \text{if }\nu \in \left[\begin{array}{cc} 0.1 & 0.2 \end{array}\right]\\ 1, & \text{if }\nu \in \left[\begin{array}{cc} 0.2 & 1.9 \end{array}\right]\\ \left(\left(2-\nu \right)/0.1\right), & \text{if }\nu \in \left[\begin{array}{cc} 1.9 & 2 \end{array}\right]\\ 0, & \text{otherwise} \end{array}\right\}$, $\mu_{\text{MRI }-\text{ tumor size }-\text{ medium}}\left(\nu \right)=\left\{\begin{array}{cc} 1, & \text{if }\nu \in \left[\begin{array}{cc} 2 & 4.9 \end{array}\right]\\ \left(\left(\nu -1.9\right)/0.1\right), & \text{if }\nu \in \left[\begin{array}{cc} 1.9 & 2 \end{array}\right]\\ \left(\left(5-\nu \right)/0.1\right), & \text{if }\nu \in \left[\begin{array}{cc} 4.9 & 5 \end{array}\right]\\ 0, & \text{otherwise} \end{array}\right\}$, $\mu_{\text{MRI }-\text{ tumor size }-\text{ high}}\left(\nu \right)=\left\{\begin{array}{cc} 1, & \text{if }\nu \in \left[\begin{array}{cc} 5 & 7.9 \end{array}\right]\\ \left(\left(\nu -4.9\right)/0.1\right), & \text{if }\nu \in \left[\begin{array}{cc} 4.9 & 5 \end{array}\right]\\ \left(\left(8-\nu \right)/0.1\right), & \text{if }\nu \in \left[\begin{array}{cc} 7.9 & 8 \end{array}\right]\\ 0, & \text{otherwise} \end{array}\right\}$, $\mu_{\text{DBCT }-\text{ lobular carcinoma}}\left(\nu \right)=\left\{\begin{array}{cc} 1, & \text{if }\nu \in \left[\begin{array}{cc} 8 & 11 \end{array}\right]\\ \left(\left(\nu -7.9\right)/0.1\right) & \text{if }\nu \in \left[\begin{array}{cc} 7.9 & 8 \end{array}\right]\\ 0, & \text{otherwise} \end{array}\right\}$

3	Positron emission tomography (*μ*_*PET*_(*θ*))	$\mu_{\text{PET }-\text{ benign}}\left(\theta \right)=\left\{\begin{array}{cc} 1, & \text{if }\theta \in \left[\begin{array}{cc} 0 & 0.4 \end{array}\right]\\ \left(\left(0.5-\theta \right)/0.1\right) & \text{if }\theta \in \left[\begin{array}{cc} 0.4 & 0.5 \end{array}\right]\\ 0, & \text{otherwise} \end{array}\right\}$, $\mu_{{\text{PET}}_{\text{spread}}\text{\_in\_whole\_body}}\left(\theta \right)=\left\{\begin{array}{cc} \left(\left(\theta -0.4\right)|/|\left(0.5-0.4\right)\right), & \text{if }\theta \in \left[\begin{array}{cc} 0.4 & 0.5 \end{array}\right]\\ 1, & \text{if }\theta \in \left[\begin{array}{cc} 0.5 & 1 \end{array}\right]\\ 0, & \text{otherwise} \end{array}\right\}$

4	Diagnosis of breast cancer stage (*μ*_*DB* *C*_(*χ*))	$\left(\mu_{\text{DBC}-\text{stage }1}\left(\chi \right)\right)=\left\{\begin{array}{cc} 1, & \text{if }\chi \in \left[\begin{array}{cc} 0 & 0.2 \end{array}\right]\\ \left(\left(0.3-\chi \right)|/|\left(0.3-0.2\right)\right), & \text{if }\chi \in \left[\begin{array}{cc} 0.2 & 0.3 \end{array}\right]\\ 0, & \text{otherwise} \end{array}\right\}$, $\left(\mu_{\text{DBC}-\text{stage }2}\left(\chi \right)\right)=\left\{\begin{array}{cc} \left(\left(\chi -0.2\right)|/|\left(0.3-0.2\right)\right), & \text{if }\chi \in \left[\begin{array}{cc} 0.2 & 0.3 \end{array}\right]\\ 1, & \text{if }\chi \in \left[\begin{array}{cc} 0.3 & 0.4 \end{array}\right]\\ \left(\left(0.5-\chi \right)|/|\left(0.5-0.4\right)\right), & \text{if }\chi \in \left[\begin{array}{cc} 0.4 & 0.5 \end{array}\right]\\ 0, & \text{otherwise} \end{array}\right\}$, $\left(\mu_{\text{DBC}-\text{stage }3}\left(\chi \right)\right)=\left\{\begin{array}{cc} \left(\left(\chi -0.4\right)|/|\left(0.5-0.4\right)\right), & \text{if }\chi \in \left[\begin{array}{cc} 0.4 & 0.5 \end{array}\right]\\ 1, & \text{if }\chi \in \left[\begin{array}{cc} 0.5 & 0.6 \end{array}\right]\\ \left(\left(0.7-\chi \right)|/|\left(0.7-0.6\right)\right), & \text{if }\chi \in \left[\begin{array}{cc} 0.6 & 0.7 \end{array}\right]\\ 0, & \text{otherwise} \end{array}\right\}$, $\left(\mu_{\text{DBC}-\text{stage }4}\left(\chi \right)\right)=\left\{\begin{array}{cc} \left(\left(\chi -0.6\right)|/|\left(0.7-0.6\right)\right), & \text{if }\chi \in \left[\begin{array}{cc} 0.6 & 0.7 \end{array}\right]\\ 1, & \text{if }\chi \in \left[\begin{array}{cc} 0.7 & 0.8 \end{array}\right]\\ \left(\left(0.9-\chi \right)|/|\left(0.9-0.8\right)\right), & \text{if }\chi \in \left[\begin{array}{cc} 0.8 & 0.9 \end{array}\right]\\ 0, & \text{otherwise} \end{array}\right\}$, $\left(\mu_{\text{DBC}-\text{stage }5}\left(\chi \right)\right)=\left\{\begin{array}{cc} \left(\left(\chi -0.8\right)|/|\left(0.9-0.8\right)\right), & \text{if }\chi \in \left[\begin{array}{cc} 0.8 & 0.9 \end{array}\right]\\ 1, & \text{if }\chi \in \left[\begin{array}{cc} 0.9 & 1 \end{array}\right]\\ 0, & \text{otherwise} \end{array}\right\}$

**Table 5:** Lookup table for layer 4 for BCP-T1F expert system.

Rules	MRI	CT	PET	Results
1	NT	N1	SHB	Severe infection
2	NT	N2	SHB	
3	NT	N3	SHB	
4	NT	N1	BEN	Stage 0
5	NT	N2	BEN	
6	LS	N1	BEN	Stage 1
7	LS	N2	BEN	
8	LS	N2	SHB	Stage 2
9	LS	N3	SHB	
10	HS	N1	BEN	Stage 3
11	HS	N3	BEN	
12	VHS	N2	BEN	Stage 4
13	VHS	N3	SHB	
14	HS	N1	SHB	
15	HS	N3	SHB	

**Table 6:** Training of the proposed BCP-SVM system model during the prediction of breast cancer.

Proposed BCP-SVM system model (70% of sample data in training)			
Total number of samples (*N* = 399)		Result (output) (*O*_*B*_ , *O*_*M*_)	
Input	Expected output (*E*_*B*_, *E*_*M*_)	*O*_*B*_ (breast cancer) positive	*O*_*M*_ (benign) negative
	*E*_*B*_ = 250 positive	248	2
	*E*_*M*_ = 149 negative	5	144

**Table 7:** Validation of the proposed BCP-SVM system model during the prediction of breast cancer.

Proposed BCP-SVM system model (30% of sample data in validation)			
Total number of samples (*N* = 170)		Result (output) (*O*_*B*_, *O*_*M*_)	
Input	Expected output (*E*_*B*_, *E*_*M*_)	*O*_*B*_ (breast cancer) positive	*O*_*M*_ (benign) negative
	*E*_*B*_ = 107 positive	106	1
	*E*_*M*_ = 63 negative	4	59

**Table 8:** Performance evaluation of proposed BCP-SVM system model in validation and training using different statistical measures.

	Sensitivity	Specificity	Accuracy	Miss rate (%)	False positive value	False negative value	Likelihood ratio positive	Likelihood ratio negative	Positive prediction value	Negative prediction value
Training	(0.9863) 98.63%	(0.9802) 98.02%	(0.9825) 98.25%	1.75	(0.0198) 1.98%	(0.0137) 1.37%	49.81	0.0139	(0.9664) 96.64%	(0.992) 99.2%
Validation	(0.9833) 98.33%	(0.9636) 96.36%	(0.9706) 97.06%	2.94	(0.0364) 3.64%	(0.0167) 1.67%	27.01	0.0173	(0.9365) 93.65%	(0.9906) 99.06%

**Table 9:** Comparison results of the proposed BCP-T1F and BCP-SVM system with literature.

Literature	Training	
	Accuracy (%)	Miss rate (%)
ANN [19]	91.10	8.9
BCP ANN [8]	92.10	7.90
ANN [11]	94	6.0
ANN-ELM [8]	96.40	3.6
ANN [17]	91.1	8.9
Proposed BCP-T1F	96.56	3.44
Proposed BCP-SVM	97.06	2.94

## Data Availability

The simulation data used to support the findings of this study are available from the corresponding author upon request.

## References

[1] Bundred N. J. (2001). Prognostic and predictive factors in breast cancer. *Cancer Treatment Reviews*.

[2] Osareh A., Shadgar B. Machine learning techniques to diagnose breast cancer.

[3] Cox D. R., Oakes D. (1984). *Analysis of Survival Data*.

[4] Brenner H., Gefeller O., Hakulinen T. (2002). A computer program for period analysis of cancer patient survival. *European Journal of Cancer*.

[5] Maglogiannis I., Zafiropoulos E., Anagnostopoulos I. (2009). An intelligent system for automated breast cancer diagnosis and prognosis using SVM based classifiers. *Applied Intelligence*.

[6] Gerasimova-Chechkina E., Toner B., Marin Z. (2016). Combining multifractal analyses of digital mammograms and infrared thermograms to assist in early breast cancer diagnosis. *AIP Conference Proceedings*.

[7] Subashini T. S., Ramalingam V., Palanivel S. (2009). Breast mass classification based on cytological patterns using RBFNN and SVM. *Expert Systems with Applications*.

[8] Prasetyo C., Kardiana A., Yuliwulandari R. (2014). Breast cancer diagnosis using artificial neural networks with extreme learning techniques. *International Journal of Advanced Research in Artificial Intelligence*.

[9] American Cancer Society (2007). *Breast Cancer Facts & Figures*.

[10] Siegel R. L., Miller K. D., Jemal A. (2017). Cancer statistics, 2017. *CA: A Cancer Journal for Clinicians*.

[11] Setiono R. (1996). Extracting rules from pruned neural networks for breast cancer diagnosis. *Artificial Intelligence in Medicine*.

[12] Tsang I. W., Kwok J. T., Cheung P. M. (2005). Core vector machines: fast SVM training on very large data sets. *Journal of Machine Learning Research*.

[13] Wolberg W. H., Mangasarian O. L. (1990). Multisurface method of pattern separation for medical diagnosis applied to breast cytology. *Proceedings of the National Academy of Sciences*.

[14] Goulding N. R., Marquez J. D., Prewett E. M. (2008). Ultrasonic imaging techniques for breast cancer detection. *AIP Conference Proceedings*.

[15] https://www.kaggle.com/uciml/breast-cancer-wisconsin-data

[16] Suckling J. P. (1994). The mammographic image analysis society digital mammogram database. *Exerpta Medica*.

[17] Brause R. W. Medical analysis and diagnosis by neural networks.

[18] Tariq N. (2017). Breast cancer detection using artificial neural network. *Journal of Molecular Biomarkers and Diagnosis*.

[19] Crockett K., Bandar Z., Mclean D., O’Shea J. (2006). On constructing a fuzzy inference framework using crisp decision trees. *Fuzzy Sets and Systems*.

[20] Khan M. U., Choi J. P., Shin H., Kim M. Predicting breast cancer survivability using fuzzy decision trees for personalized healthcare.

[21] Sison L. G., Chong E. K. Fuzzy modeling by induction and pruning of decision trees.

[22] Umano M., Okamoto H., Hatano I. Generation of fuzzy decision trees by fuzzy ID3 algorithm and its application to diagnosis by gas in oil.

[23] Ian H., Witten, Eibe Frank (2005). *Data Mining: Practiacal Machine Learning Tools and Techniques*.

[24] Nurhasanah, Sampurno J., Faryuni I. D., Ivansyah O. (2016). Automated analysis of image mammogram for breast cancer diagnosis. *AIP Conference Proceedings*.

[25] Wadhwani S., Singh T., Singh Bhadauoria S. (2012). BCD-clustering algorithm for breast cancer diagnosis. *International Journal of Scientific and Research Publications*.

[26] Balanică V., Dumitrache I., Caramihai M., Rae W., Herbst C. (2011). Evaluation of breast cancer risk by using fuzzy logic. *University Politehnica of Bucharest Scientific Bulletin, Series C*.

[27] Fogel D. B., Wasson E. C., Boughton E. M., Porto V. W. (1998). Evolving artificial neural networks for screening features from mammograms. *Artificial Intelligence in Medicine*.

[28] Bellazzi R., Ironi L., Guglielmann R., Stefanelli M. (1998). Qualitative models and fuzzy systems: an integrated approach for learning from data. *Artificial Intelligence in Medicine*.

[29] Bagui S. C., Bagui S., Pal K., Pal N. R. (2003). Breast cancer detection using rank nearest neighbor classification rules. *Pattern Recognition*.

[30] Bellazzi R., Guglielmann R., Ironi L. (2003). Qualitative models and fuzzy systems: an integrated approach to system identification. *Soft Computing Applications*.

[31] Miao G. J., Miao K. H., Miao J. H. (2012). Neural pattern recognition model for breast cancer diagnosis. *Journal of Selected Areas in Bioinformatics*.

[32] Torres A., Nieto J. J. (2006). Fuzzy logic in medicine and bioinformatics. *Journal of Biomedicine and Biotechnology*.

[33] Cristianini N., Kandola J., Elisseeff A., Shawe-Taylor J. (2001). On optimizing Kernel alignment. http://www.iipl.fudan.edu.cn/~zhangjp/literatures/cluster%20analysis/01087.ps.

[34] Huang G. B., Babri H. A. (1998). Upper bounds on the number of hidden neurons in feedforward networks with arbitrary bounded nonlinear activation functions. *IEEE Transactions on Neural Networks*.

[35] Singh A. K., Gupta B. (2015). A novel approach for breast cancer detection and segmentation in a mammogram. *Procedia Computer Science*.

[36] Dheeba J., Albert Singh N., Tamil Selvi S. (2014). Computer-aided detection of breast cancer on mammograms: a swarm intelligence optimized wavelet neural network approach. *Journal of Biomedical Informatics*.

[37] Hussain S., Abbas S., Sohail T., Adnan Khan M., Athar A. (2019). Estimating virtual trust of cognitive agents using multi layered socio-fuzzy inference system. *Journal of Intelligent & Fuzzy Systems*.

[38] Fatima A., Khan M. A., Abbas S., Waqas M., Anum L., Asif M. (2019). Evaluation of planet factors of smart city through multi-layer fuzzy logic (MFL). *The ISC International Journal of Information Security*.

[39] Siddiqui S. Y., Hussnain S. A., Siddiqui A. H. (2019). Diagnosis of arthritis using adaptive hierarchical Mamdani fuzzy type-1 expert system. *ICST Transactions on Scalable Information Systems*.

[40] Atta A., Abbas S., Khan M. A., Ahmed G., Farooq U. (2018). An adaptive approach: smart traffic congestion control system. *Journal of King Saud University-Computer and Information Sciences*.

[41] Khan M. A., Umair M., Saleem M. A., Ali M. N., Abbas S. (2019). CDE using improved opposite based swarm optimization for MIMO systems. *Journal of Intelligent & Fuzzy Systems*.

[42] Khan M. A., Umair M., Choudhry M. A. S. (2015). GA based adaptive receiver for MC-CDMA system. *Turkish Journal of Electrical Engineering & Computer Sciences*.

[43] Khan M. A., Umair M., Choudry M. A. S. Island differential evolution based adaptive receiver for MC-CDMA system.

[44] Ali M. N., Khan M. A., Adeel M., Amir M. (2016). Genetic algorithm based adaptive receiver for MC-CDMA system with variation in mutation operator. *International Journal of Computer Science and Information Security*.

[45] Umair M., Khan M. A., Choudry M. A. S. Island genetic algorithm based MUD for MC-CDMA system.

[46] Umair M., Khan M. A., Choudry M. A. S. GA backing to STBC based MC-CDMA systems.

[47] Kashif I., Muhammad A. K., Sagheer A., Zahid H., Areej F. (2018). Intelligent transportation system (ITS) for smart-cities using Mamdani fuzzy inference system. *International Journal of Advanced Computer Science and Applications (IJACSA)*.

[48] Alqudah A. M., Algharib H. M. S., Algharib A. M. S., Algharib H. M. S. (2019). Computer aided diagnosis system for automatic two stages classification of breast mass in digital mammogram images. *Biomedical Engineering: Applications, Basis and Communications*.

[49] Alqudah A., Alqudah A. M. (2019). Sliding window based support vector machine system for classification of breast cancer using histopathological microscopic images. *IETE Journal of Research*.

[50] Wang H., Zhang Z., Taleb T. (2018). Editorial: special issue on security and privacy of IoT. *World Wide Web*.

[51] MathWorks, Inc (2017). MATLAB documentation. https://www.mathworks.com/help/matlab/index.html?s_tid=gn_loc_drop.

